# Reply to ‘Commentary on: Mapping the landscape of immunonutrition and cancer research: a comprehensive bibliometric analysis on behalf of NutriOnc Research Group (Int J Surg 2023, Epub ahead of print)’

**DOI:** 10.1097/JS9.0000000000001300

**Published:** 2024-03-15

**Authors:** Francesca De Felice, Carlo G. Cattaneo, Luigi Marano

**Affiliations:** a Department of Radiotherapy, Policlinico Umberto I, Department of Radiological, Oncological and Pathological Sciences, “Sapienza” University of Rome, Rome, Italy; b Department of Medicine, Academy of Applied Medical and Social Sciences, Akademia Medycznych I Spolecznych Nauk Stosowanych (AMiSNS), Elbląg; c Department of General Surgery and Surgical Oncology, “Saint Wojciech” Hospital, “Nicolaus Copernicus” Health Center, Gdańsk, Poland

*Dear Editor*,

We would like to thank Wang *et al*.^1^ for their insightful comments regarding our recent publication, ‘Mapping the landscape of immunonutrition and cancer research: a comprehensive bibliometric analysis on behalf of NutriOnc Research Group’^2^. We also appreciate the acknowledgment of the central role of nutritional support in oncology.

We understand the concerns raised regarding the methodology employed in our bibliometric analysis. While our primary keywords were ‘immunonutrition’ and ‘cancer,’ it is true that common immunonutrients such as glutamate, arginine, omega-3 polyunsaturated fatty acids, and nucleotides were not explicitly mentioned. However, we would like to clarify that our intention was not to exclude studies focusing on these specific nutrients but rather to capture a broad overview of research trends in immunonutrition applied to cancer patients. Our choice of keywords was guided by the aim to provide a comprehensive analysis while minimizing the risk of overlooking relevant studies. We recognize the importance of these specific nutrients in immunonutrition and acknowledge that future studies could benefit from their explicit inclusion in the search strategy. Additionally, we appreciate the point raised regarding the type of literature included in our analysis. While our manuscript did not explicitly mention the type of literature included, we would like to clarify that our search was focused on peer-reviewed articles published in academic journals. We agree with the importance of screening the type of literature included in bibliometric analyses to ensure the quality and relevance of the findings.

Regarding the timeframe selection from 1 January 1998, to 15 May 2023, we aim to provide further context regarding our decision-making process. Our choice of timeframe was guided by several considerations, including the need to capture a comprehensive overview of immunonutrition research applied to cancer management while ensuring the feasibility of data collection and analysis. The decision to commence our analysis from 1 January 1998, was based on the availability of digital databases and the increasing emphasis on immunonutrition in clinical research during the late 1990s. Importantly, our preliminary analysis included a thorough examination of available literature, and we made a deliberate decision to include studies published within the specified timeframe to maintain consistency and coherence in our analysis. By selecting studies from the same cohort but updating them to include the most recent publications, we aimed to provide an updated and comprehensive assessment of emerging trends and patterns in the field of immunonutrition and cancer research.

Furthermore, while our study acknowledges the categorization of scientific research into various stages, we firmly assert that the initial stage of the field is not incomplete within the context of our analysis. Our study provides valuable insights into the evolution of immunonutrition research over the past 25 years, capturing the contemporary snapshot that has laid the groundwork for current advancements in the field.

Finally, we welcome the opportunity to address the concerns raised regarding the lack of quality assessment in our study. We respectfully disagree with the assertion that it is a missing component in bibliometric studies. While we acknowledge the significance of data quality control in research, our study aimed to provide a macroscopic overview of emerging trends and patterns in immunonutrition applied to cancer research through bibliometric analysis. Our focus was on analyzing publication trends, identifying research hotspots, and mapping collaboration networks within the field. The primary objective of our study was to provide a comprehensive overview rather than conduct an in-depth quality assessment of individual studies. Furthermore, it is worth noting that the BIBLIO checklist for reporting bibliometric reviews of the biomedical literature, as published in the ‘Preliminary guideline for reporting bibliometric reviews of the biomedical literature (BIBLIO): a minimum requirements’^3^, does not indicate the quality assessment of selected studies as an obligatory requirement. As such, our study adhered to the established reporting guidelines for bibliometric analyses within the biomedical literature.

In conclusion, we extend our gratitude for the constructive commentary and reaffirm our dedication to advancing the understanding of immunonutrition in cancer research through rigorous methodology.

## Ethical approval

No ethical approval is needed as it is Reply to commentary.

## Consent

No consent was obtained as it is a Reply to commentary.

## Sources of funding

The authors declare no source of funding.

## Author contribution

F.D.F., C.G.C., and L.M.: conceptualization, project administration, supervision, writing – original draft, and writing – review and editing.

## Conflicts of interest disclosure

The authors declare no conflicts of interest.

## Research registration unique identifying number (UIN)

It is a Reply to commentary.

## Guarantor

The guarantor is Prof Luigi Marano.

## Data availability statement

Not applicable.

## Provenance and peer review

Not commissioned.
